# Optimization of double diffusive mixed convection in a BFS channel filled with Alumina nanoparticle using Taguchi method and utility concept

**DOI:** 10.1038/s41598-019-55897-y

**Published:** 2019-12-20

**Authors:** Ratnadeep Nath, Murugesan Krishnan

**Affiliations:** 1 Research scholar, Mechanical & Industrial Engineering Department, Indian Institute of Technology Roorkee, Roorkee, 247 667 India; 2 Professor, Mechanical & Industrial Engineering Department, Indian Institute of Technology Roorkee, Roorkee, 247 667 India

**Keywords:** Mechanical engineering, Nanoscale materials

## Abstract

This research work focuses on the implementation of Taguchi method and utility concept for optimization of flow, geometrical and thermo-physical parameters for mixed convective heat and mass transfer in a backward facing step (BFS) channel filled with Alumina nanoparticle doped in water-ethylene glycol mixture. Mass, momentum, energy and solutal conservation equations for the flow field are cast in velocity-vorticity form of Navier-Stokes equations, which are solved using Galerkin’s weighted residual finite element method through isoparametric formulation. The following six parameters, expansion ratio of the BFS channel (H/h), Reynolds number (Re), buoyancy ratio (N), nanoparticle volume fraction (χ), shape of nanoparticles and thermal Grashof number (Gr_T_) at three levels are considered as controlling parameters for optimization using Taguchi method. An L_27_ orthogonal array has been chosen to get the levels of the six parameters for the 27 trial runs. Simulation results were obtained for 27 trial runs from which three different sets of optimum levels of the control parameters were obtained for maximum Nu and Sh and minimum wall shear stress during double diffusive mixed convection in the channel. Then, in order to obtain a single set of optimum levels of the control parameters to achieve maximum heat and mass transfer and minimum wall shear stress concurrently, utility concept has been implemented. Taguchi results indicate that expansion ratio and volume fraction of nanoparticles are the significant contributing parameters to achieve maximum heat and mass transfer and minimum wall shear stress. Utility concept predicts the average Nusselt number less by 2% and Sherwood number less by 3% compared to the Taguchi method with equal weightage of 40% assumed for Nusselt and Sherwood numbers and 20% for wall shear stress.

## Introduction

Nanofluid technology is a well-established method for heat transfer enhancement in convective heat transfer cooling applications^1^. Heat transfer in nanofluids for natural^2,3^, forced^4,5^ and mixed convection^6–8^ phenomena have been studied in detail to understand the effect of flow and heat transfer parameters such as Re, Ri, Grashof number on heat transfer. Doping of nanoparticles in the base fluid essentially modifies the thermo-physical properties of the fluid which influence the flow pattern and heat transport mechanism in a given geometry. Reddy and Murugesan^9,10^ analyzed heat and mass transport in square enclosures to understand the effect of aiding and opposing buoyancy forces on heat transfer phenomena while Mohammed *et al*.^11^ and Nath and Krishnan^12^ investigated backward facing step (BFS) channel to analyze the behavior of nanofluid on flow separation and re-attachment length under aiding and opposing buoyancy forces. In addition to the flow and geometrical parameters, researchers have also focused on the study of different shape of nanoparticles other than the commonly used spherical type nanoparticles^13^. Nanoparticles of different shapes exhibit distinct behavior on the variation of thermal conductivity and viscosity of the resulting nanofluid when doped with the base fluid. Ferrouillat *et al*.^14^ experimentally analyzed the effect of shape of nanoparticle on convective heat transfer phenomena and found that water-ZnO nanofluid with polygonal shape showed 8% increment in Nu while rod like shape showed 3% increment in Nu and this difference was attributed to the dynamic viscosity of the nanofluid. Similarly, other shapes of nanoparticle such as spheroids, prolate spheroids^15,16^, platelet, bricks, blades, cylindrical^13^ were also considered by researchers to study their effect on heat and mass transfer. It is found that geometrical properties of the cavity/channel, flow parameters and thermo-physical properties of the working fluid influence the variation in nature and magnitude of heat and mass transfer and wall shear stress for problems involving flow separation. In order to achieve efficient design of equipment that use nanofluid as heat transfer fluids, it is essential to determine the optimum values of the influencing parameters for the given flow situation. Using parametric study, results for many combinations of a number of parameters could be obtained, however, it will be highly challenging to analyze the vast amount of results in order to obtain optimum values of the parameters. Such situations can be handled scientifically by employing most reliable optimization tools. Optimization techniques such as generic algorithm (GA), sequential quadratic programming (SQP), fuzzy logic, artificial neural network (ANN), response surface methodology (RSM) and Taguchi method etc. are some of the optimization techniques that are well established in engineering applications.

Taguchi method is widely used as a robust cost effective technique that works on the principle of Design of Experiment (DoE) and is well established in the field of product quality and reliability^17–19^. Although, Taguchi-a Japanese technique was originally used to improve the quality of manufacturing goods, later scientists successfully implemented for heat transfer problems as well. Three type of optimization criteria have been defined by the Taguchi method, ‘larger the better’, ‘smaller the better’ and ‘nominal the better’. As per the requirement in a given problem, one of these criteria can be used to compute the signal to noise (S/N) ratio, which will serve as a useful function to estimate the contribution of various influencing parameters on the variables being optimized. Scientists used this technique in various thermal engineering application such as cooling problems^20,21^, solar energy applications^22,23^ and nanofluid characteristics^24,25^ etc. Jamshidi *et al*.^26^ used helical coils filled with Al_2_O_3_-water nanofluid and optimized the design parameters using Taguchi method. The authors considered ‘higher the better’ concept for j-f factor and made use of L_9_ orthogonal array with three levels of the control parameters. Nanofluid with temperature dependent property shows optimum condition and thus maximum connective heat transfer was achieved with minor penalty of fiction factor. Mamourian *et al*.^27^ and Shirvan *et al*.^28^ maximized the Nusselt number for mixed convective heat transfer in an enclosure filled with a nanofluid by optimizing the control parameters using Taguchi technique. They found that Ri number is a significant parameter to achieve maximum heat transfer. Recently, Sobhani and Ajam^29^ carried out a similar Taguchi analysis in a cavity for natural convection heat transfer with Al_2_O_3_ nanoparticle. The authors considered five control parameters selected from heat transfer parameters and volume fraction of nanoparticle at three levels to conduct 27 experimental trials using L_27_ orthogonal array. The authors noticed that Rayleigh number contributes 80.42% for heat transfer, thus supporting the underlying physics of natural convection. Abdollahi and Shams^30^ focused on multi-objective optimization in a nanofluid filled channel with vortex generator considering two objective functions, maximization of Nusselt number and minimization of friction factor using ANN. The authors also employed Non-dominated Sorting Generic Algorithm - II (NSGA-II) and found that the trapezoidal vortex generator gave the best thermo-hydraulic performance using 0.3% of nanoparticle volume fraction with 51° attack angle. It is to be noted that the above algorithms are not capable of presenting the ANOVA table from which contribution of each parameter can be obtained.

From a detailed literature study, it is found that study of heat and mass transfer in BFS channel problems with nanofluid has attracted many researchers in respect of the effect of different type of nanoparticles, different shape of nanoparticles, volume fraction of nanoparticles, Richardson number, geometrical parameters etc. With an aim to achieve maximum heat transfer, researchers have implemented different optimization techniques including Taguchi method in order to determine the optimum set of parameters. Though a number of research articles have contributed on optimizing the flow and geometrical parameters, still there is a research gap for detailed study for both heat and mass transfer in BFS channel problems. All the research works have considered optimization of heat transfer without considering multi-objective functions due to the limitation of the optimization techniques employed. Simultaneous heat and mass transfer problems produce many interesting phenomena when both thermal and solutal buoyancy forces are considered either in aiding or in opposing modes. In addition to this, the combination of nanoparticle characteristics such as volume fraction and shape will add value to the research problem bringing out many interesting research insights. Thus in the present research work, an attempt has been made to address this research gap by carrying out a detailed optimization analysis of double diffusive mixed convection problem in a BFS channel filled with nanofluid. Three objective functions, maximization of heat transfer, maximization of mass transfer and minimization of wall shear stress are considered for optimization. For this purpose, initially Taguchi method has been employed to determine the levels of control variables for each objective function separately and then utility concept has been implemented to estimate the levels of the control parameters by satisfying all the three objective functions concurrently. For this purpose, six parameters are chosen from geometry, flow, nanoparticle characteristics and heat transfer characteristic, such as, expansion ratio of the channel, Reynolds number, buoyancy ratio, nanoparticle volume fraction, shape of nanoparticle and thermal Grashof number. The details of the implementation of the techniques and discussion on the results obtained are explained in the following sections.

## Problem Description

**Figure 1:**
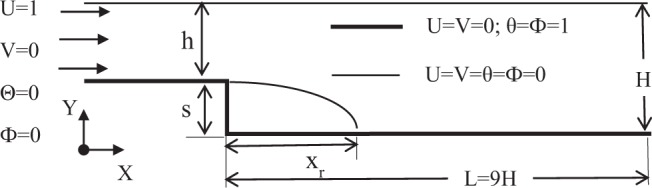
Schematic diagram of a backward facing step (BFS) channel.

The present research work focuses on optimizing the flow and heat transfer parameters for double diffusive mixed convection with nanofluid in a backward facing step (BFS) channel that is shown in Fig. 1. The step height (s), inlet channel height (h), outlet channel height (H), expansion ratio (H/h) and length of channel is L (= 9 H). The flow at the entrance of the channel is considered to be hydrodynamically steady and uniform. It is further assumed that the flow is incompressible, Newtonian and two dimensional. The working fluid consists of alumina (Al_2_O_3_) nanoparticle mixed in water and ethylene glycol (EG) of equal volume (50–50%) where nanoparticle and base fluid are assumed to be in thermal equilibrium with no slip condition. Thermo-physical properties of alumina and water/EG are given in Table 1. It is assumed that the operating range of base fluid (water-EG) is about 25–30 °C and thus the Prandtl number (Pr) is set as 6.2. It is further assumed that solutal diffusivity is as strong as thermal diffusivity, thus making Lewis number (Le) equal to 1.0. The entire bottom wall is kept at high temperature (T_h_), high concentration (C_h_) whereas the top wall is maintained at lower values of temperature and concentration (T_c_, C_c_). A cold and low concentrated fluid is assumed to enter into the BFS channel. The presence of thermo-solutal gradient causes density variation of the fluid and can be simplified using Boussinesq approximation and can be expressed as ρ = ρ_0_ [1 − β_T,nf_ (T − T_c_) − β_C,nf_ (C − C_c_)], where ρ denotes the density, β_T_ & β_C_ are co-efficient of thermal and solutal expansion, nf denotes nanofluid. Co-efficient of thermal expansion is always positive whereas for solutal expansion it is either positive or negative depending upon the fluid density variation and solutal concentration. Hence, depending upon the type of use of solutal particle the solutal buoyancy force may aid or oppose the thermal buoyancy force.

**Table 1:** Thermophysical properties of nanofluid.

Properties	Water (base fluid)	Ethylene glycol (base fluid)	Al_2_O_3_
c_p_ (J/kg K)	4182.2	2415	765
*ρ* (kg/m^3^)	998.203	1114.4	3970
k (w/m K)	0.613	0.252	40
*β*_*T*_ × 10^−4^(1/K)	2.06	6.50	0.08

### Governing equations and solution procedure

The physics of fluid flow and heat and mass transfer in the channel is governed by the conservation principles such as mass, momentum, energy and solutal concentration for the flow field with nanoparticles and are represented in non-dimensional velocity-vorticity form as follows:

Vorticity transport equation:

1 $$\frac{\partial {\rm{\zeta }}}{\partial {\rm{\tau }}}+{\rm{U}}\frac{\partial {\rm{\zeta }}}{\partial {\rm{X}}}+V\frac{\partial {\rm{\zeta }}}{\partial {\rm{Y}}}=\frac{1}{{\rm{Re}}}\frac{(1+{{\rm{A}}}_{1}{\rm{\chi }}+{{\rm{A}}}_{2}{{\rm{\chi }}}^{2})}{((1-{\rm{\chi }})+\frac{{{\rm{\chi }}{\rm{\rho }}}_{{\rm{p}}}}{{{\rm{\rho }}}_{{\rm{f}}}})}(\frac{{\partial }^{2}{\rm{\zeta }}}{\partial {{\rm{X}}}^{2}}+\frac{{\partial }^{2}{\rm{\zeta }}}{\partial {{\rm{Y}}}^{2}})+\frac{{{\rm{Gr}}}_{{\rm{T}}}}{{{\rm{Re}}}^{2}}[\frac{(1-{\rm{\chi }}){{\rm{\rho }}}_{{\rm{f}}}+\frac{{{\rm{\chi }}{\rm{\rho }}}_{{\rm{p}}}{{\rm{\beta }}}_{{{\rm{T}}}_{{\rm{p}}}}}{{{\rm{\rho }}}_{{\rm{f}}}{{\rm{\beta }}}_{{{\rm{T}}}_{{\rm{f}}}}}}{(1-{\rm{\chi }}){{\rm{\rho }}}_{{\rm{f}}}+{{\rm{\chi }}{\rm{\rho }}}_{{\rm{p}}}}]\frac{\partial }{\partial {\rm{X}}}({\rm{\theta }}+{\rm{N}}\Phi )$$

where, ζ is non-dimensional vorticity $(\frac{{\rm{\omega }}{\rm{H}}}{{{\rm{U}}}_{0}})$, *τ* is non-dimensional time $(\frac{{{\rm{tU}}}_{0}}{{\rm{H}}})$, X and Y are non-dimensional space coordinates $(\frac{{\rm{x}}}{{\rm{H}}},\frac{{\rm{y}}}{{\rm{H}}})$, U and V are non-dimensional velocity components $(\frac{{\rm{u}}}{{{\rm{U}}}_{0}},\frac{{\rm{v}}}{{{\rm{U}}}_{0}})$, Re is Reynolds number $(\frac{{{\rm{U}}}_{0}{\rm{H}}}{{{\rm{\gamma }}}_{{\rm{f}}}})$, Gr_T_ is Grashof number due to thermal diffusion $(\frac{{{\rm{g}}{\rm{\beta }}}_{{{\rm{T}}}_{{\rm{f}}}}{\Delta {\rm{TH}}}^{3}}{{{\rm{\gamma }}}_{{\rm{f}}}^{2}})$, N is buoyancy ratio $(\frac{{{\rm{\beta }}}_{{{\rm{C}}}_{{\rm{nf}}}}\Delta {\rm{C}}}{{{\rm{\beta }}}_{{{\rm{T}}}_{{\rm{nf}}}}\Delta {\rm{T}}})$, χ is nanoparticle volumetric fraction, p and f denote solid nanoparticle and base fluid. The dimensional variables used to form non-dimensional terms are such as *ω* is vorticity vector, x and y are dimensional space coordinates, t is dimensional time, u and v are dimensional velocity components, U_0_ is the inlet flow velocity, g is the gravity, γ is the kinematic viscosity.

The vorticity *ζ* have two unknown components of velocity vector. Hence, it requires another equation i.e. velocity Poisson equation to solve the flow field.

Velocity Poisson equation:

2a $$\frac{{\partial }^{2}{\rm{U}}}{\partial {{\rm{X}}}^{2}}+\frac{{\partial }^{2}{\rm{U}}}{\partial {{\rm{Y}}}^{2}}=-\,\frac{\partial {\rm{\zeta }}}{\partial {\rm{Y}}}$$

2b $$\frac{{\partial }^{2}{\rm{V}}}{\partial {{\rm{X}}}^{2}}+\frac{{\partial }^{2}{\rm{V}}}{\partial {{\rm{Y}}}^{2}}=\frac{\partial \zeta }{\partial {\rm{X}}}$$

Energy equation:

3 $$\frac{\partial {\rm{\theta }}}{\partial {\rm{\tau }}}+{\rm{U}}\frac{\partial {\rm{\theta }}}{\partial {\rm{X}}}+{\rm{V}}\frac{\partial {\rm{\theta }}}{\partial {\rm{Y}}}=\frac{1}{{\rm{Re}}.\,{\rm{\Pr }}}\frac{(1+{{\rm{C}}}_{{\rm{k}}}{\rm{\chi }})}{((1-{\rm{\chi }})+{\rm{\chi }}\frac{{({{\rm{\rho }}{\rm{c}}}_{{\rm{p}}})}_{{\rm{p}}}}{{({{\rm{\rho }}{\rm{c}}}_{{\rm{p}}})}_{{\rm{f}}}})}(\frac{{\partial }^{2}{\rm{\theta }}}{\partial {{\rm{X}}}^{2}}+\frac{{\partial }^{2}{\rm{\theta }}}{\partial {{\rm{Y}}}^{2}})$$

where, *θ* is non-dimensional temperature $(\frac{{\rm{T}}-{{\rm{T}}}_{{\rm{c}}}}{{{\rm{T}}}_{{\rm{h}}}-{{\rm{T}}}_{{\rm{c}}}})$, Pr is Prandtl number $(\frac{{{\rm{\gamma }}}_{{\rm{f}}}}{{{\rm{\alpha }}}_{{\rm{f}}}})$, c_p_ is the specific heat, *α* is the thermal diffusivity.

Solutal concentration equation:

4 $$\frac{\partial \Phi }{\partial {\rm{\tau }}}+{\rm{U}}\frac{\partial \Phi }{\partial {\rm{X}}}+{\rm{V}}\frac{\partial \Phi }{\partial {\rm{Y}}}=\frac{(1-{\rm{\chi }})}{{\rm{Re}}.{\rm{Sc}}}(\frac{{\partial }^{2}\Phi }{\partial {{\rm{X}}}^{2}}+\frac{{\partial }^{2}\Phi }{\partial {{\rm{Y}}}^{2}})$$

where, Ф is the non-dimensional concentration $(\frac{{\rm{C}}-{{\rm{C}}}_{{\rm{c}}}}{{{\rm{C}}}_{{\rm{h}}}-{{\rm{C}}}_{{\rm{c}}}})$, *Sc* is the Schmidt number $(\frac{{{\rm{\gamma }}}_{{\rm{f}}}}{{{\rm{D}}}_{{\rm{f}}}})$, D is the solutal diffusion co-efficient.

Thermo-physical models of nanofluid are inculcated in the present formulation. The effective density, heat capacitance and mass diffusivity of nanofluid are taken from Nath and Krishnan^12^ paper. The thermal conductivity and dynamic viscosity for three different shapes of nanoparticle can be calculated using Timofeeva *et al*. model^31^. Constants values such as A_1_, A_2_ and C_k_ for blades, cylinders and bricks shaped nanoparticles used in Timofeeva *et al*. model^31^ are listed in Table 2.

5 $$\begin{array}{c}{\rm{I}}{\rm{n}}{\rm{i}}{\rm{t}}{\rm{i}}{\rm{a}}{\rm{l}}\,{\rm{c}}{\rm{o}}{\rm{n}}{\rm{d}}{\rm{i}}{\rm{t}}{\rm{i}}{\rm{o}}{\rm{n}}{\rm{s}}\,({\rm{a}}{\rm{t}}\,\tau =0):\\ {\rm{U}}={\rm{V}}=\theta =\Phi =0\end{array}$$

6 $${\rm{Boundary}}\,{\rm{conditions}}\,{\rm{for}}\,{\rm{the}}\,{\rm{above}}\,{\rm{set}}\,{\rm{of}}\,{\rm{governing}}\,{\rm{equations}}\,{\rm{are}}\,{\rm{as}}\,{\rm{follows}}\,({\rm{at}}\,{\rm{\tau }} > 0):$$

(i) Upstream inlet conditions: ${\rm{U}}=1,\,{\rm{V}}={\rm{\theta }}=\Phi =0$
(ii) Downstream outlet conditions: $\frac{\partial {\rm{U}}}{\partial {\rm{X}}}=\frac{\partial {\rm{V}}}{\partial {\rm{X}}}=\frac{\partial {\rm{\theta }}}{\partial {\rm{X}}}=\frac{\partial \Phi }{\partial {\rm{X}}}=0$
(iii) Top wall conditions: ${\rm{U}}={\rm{V}}={\rm{\theta }}=\Phi =0$
(iv) Bottom wall conditions: ${\rm{U}}={\rm{V}}=0;\,{\rm{\theta }}=\Phi =1$

**Table 2:** Viscosity enhancement and thermal conductivity coefficients for different shaped nanoparticles.

	Blades	Cylinders	Bricks
A_1_	14.6	13.5	1.9
A_2_	123.3	904.4	471.4
C_k_	2.74	3.95	3.37

After solving the variables i.e. U, V, θ, Ф further useful non-dimensional parameters are computed. The present study focusses on three important parameters i.e. average Nu, average Sh and average wall shear stress calculated over the bottom heated wall after the step and can be written as:

7 $${{\rm{Nu}}}_{{\rm{avg}}}=-\,\frac{1}{{\rm{L}}}\frac{{{\rm{k}}}_{{\rm{nf}}}}{{{\rm{k}}}_{{\rm{f}}}}{\int }_{0}^{{\rm{L}}}{\frac{\partial {\rm{\theta }}}{\partial {\rm{Y}}}|}_{{\rm{Y}}=0}{\rm{dX}};\,{{\rm{Sh}}}_{{\rm{avg}}}=-\,\frac{1}{{\rm{L}}}\frac{{{\rm{D}}}_{{\rm{nf}}}}{{{\rm{D}}}_{{\rm{f}}}}{\int }_{0}^{{\rm{L}}}{\frac{\partial \Phi }{\partial {\rm{Y}}}|}_{Y=0}{\rm{dX}};\,{{{\rm{\tau }}}_{{\rm{w}}}|}_{{\rm{avg}}}=\frac{1}{{\rm{L}}}\frac{{{\rm{\mu }}}_{{\rm{nf}}}}{{{\rm{\mu }}}_{{\rm{f}}}}{\int }_{0}^{{\rm{L}}}{\frac{\partial {\rm{U}}}{\partial {\rm{Y}}}|}_{Y=0}{\rm{dX}}$$

where, Nu, Sh and τ_w_ refer as Nusselt number, Sherwood number and wall shear stress; avg denotes average value.

The governing equations of momentum, energy, and concentration in Eqs. (1) to (4) are solved by applying Galerkin’s weighted residual finite element technique imposing the boundary conditions given in Eqs. (5) and (6). Using isoparametric formulation, bilinear quadrilateral elements are developed for grid generation. A second order accuracy Crank-Nicolson scheme is used to discretize the time derivative. The governing equations are finally transformed to simultaneous algebraic equations and conjugate gradient (CG) iterative solver with global matrix free algorithm^32^ is used to solve the flow variables. An in-house built FORTRAN code is developed for this purpose and it is being thoroughly used by the authors’ group. The governing equations are assumed to be converged until a steady state solution is obtained. The convergence criterion for any primary variable Γ is set to 10^−5^ which can be expressed by the following equation:

8 $$\frac{\displaystyle \mathop{\sum }\limits_{{\rm{m}}=1}^{{\rm{nnode}}}[|{\Gamma }_{{\rm{m}}}^{{\rm{n}}+1}|-|{\Gamma }_{{\rm{m}}}^{{\rm{n}}}|]}{{\rm{nnode}}}\le {10}^{-5}$$

### Optimization methodology

#### Taguchi method

**Figure 2:**
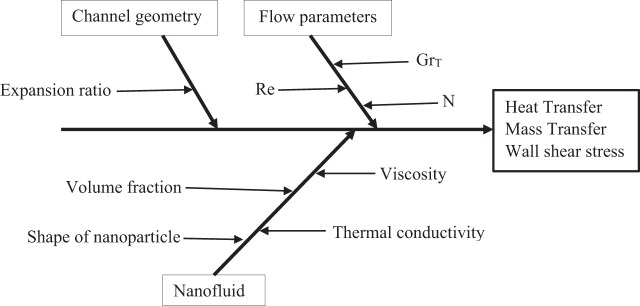
Cause & effect diagram (fishbone diagram) showing factors that have influence on heat and mass transfer and wall shear stress in a BFS channel filled with nanofluid.

Taguchi optimization method is a statistical plan of experiments that uses Latin square arrangement to form orthogonal array (OA) in matrix form of experiments. It gives optimum number (like 4, 8, 16 etc.) of experimental trial runs with control parameters and finally the best level of every single parameter is found. Figure 2 shows the cause and effect diagram (Fishbone diagram) depicting the influential parameters involved in heat and mass transfer and wall friction for flow over a BFS channel filled with a nanofluid. The method starts with the selection of control parameters and their levels for the problem under consideration. Secondly, depending upon the number of control parameters and levels an orthogonal array (for example L_4_, L_8_, L_16_) is selected and experiments are performed for each trial accordingly. Finally, using signal to noise (S/N) ratio, ANOVA and response table the results are analyzed. The performance of the response can be categorized into three different characteristics i.e. lower the better, nominal the better and higher the better. The great advantage of Taguchi method is that it is based on Design of Experiments (DoE) and its inbuilt error analysis technique. The variance analysis gives the error difference if experiments based on the selected number of trials are done actually. Percentage contribution of each parameter in the numerical experiment can be found from ANOVA table.

For a given number of control factors and levels, the optimum number of experiments to be carried out can be preset using the following expression^35,36^:

9 $${{\rm{N}}}_{{\rm{Taguchi}}}=1+{\rm{NF}}({\rm{L}}-1)$$

where, N_Taguchi_ is the minimum number of numerical experiments to be conducted, NF is the number of control factors (parameters) and L is the number of levels used. In the present study six parameters (NF = 6) and three levels (L = 3) have been considered as shown in Table 3. From the above equation, minimum 13 numerical experiments are required. However, for three level factors, only L_9_, L_27_, L_81_ etc. orthogonal arrays are available^35^. Hence, the nearest orthogonal array, L_27_ is used for the present experimental trials and the levels of six parameters for each trial run are shown in Table 4. There are three main different objectives in this present research that is to optimize the average Nusselt number and Sherwood number for which higher the better criterion has been selected and the third objective is to optimize average wall shear stress for which lower the better criterion has been selected. Using the set of levels of parameters for each experiment as given in Table 4, numerical simulation results have been obtained and average Nusselt number, Sherwood number and wall shear stress were computed for each trial run and are shown in Table 5 along with the respective S/N ratios. For higher the better and lower the better characteristics, signal to noise ratio (SNR) can be given by the following equations^37–39^:

10 $${\rm{Higher}}\,{\rm{the}}\,{\rm{better}}:{{\rm{SNR}}}_{{\rm{HB}}}=-\,10\,\log (\frac{1}{{\rm{n}}}\mathop{\sum }\limits_{{\rm{j}}=1}^{{\rm{n}}}\frac{1}{{{\rm{y}}}_{{\rm{j}}}^{2}})$$

11 $${\rm{Lower}}\,{\rm{the}}\,{\rm{better}}:{{\rm{SNR}}}_{{\rm{LB}}}=-\,10\,\log (\frac{1}{n}\mathop{\sum }\limits_{{\rm{j}}=1}^{{\rm{n}}}{{\rm{y}}}_{{\rm{j}}}^{2})$$

where, y_j_ is the response data at j^th^ number of trial. Taguchi optimization technique gives the optimum performance with a best set of parameters for a single objective function once at a time. But when a system needs an optimum performance for multi-objective functions, Taguchi method gives multiple set of control factors and hence utility concept has to be employed to get single set of control factors.

**Table 3:** Control variables and levels.

Label	Control parameters	Level		
		1	2	3
A	Expansion ratio (H/h)	1.42	2	2.5
B	Reynolds number (Re)	100	150	200
C	Buoyancy ratio (N)	−3	1	3
D	Nanoparticle volume fraction (χ)	1%	3%	5%
E	Shape of nanoparticle (shape)	Bricks	Blades	Cylinders
F	Thermal Grashof number (Gr_T_)	1000	2250	4000

**Table 4:** Taguchi L_27_ orthogonal array (OA).

Exp. No	Factor (A)	Factor (B)	Factor (C)	Factor (D)	Factor (E)	Factor (F)
1.	**1**	**1**	**1**	**1**	**1**	**1**
2.	**1**	**1**	**1**	**1**	**2**	**2**
3.	**1**	**1**	**1**	**1**	**3**	**3**
4.	**1**	**2**	**2**	**2**	**1**	**1**
5.	**1**	**2**	**2**	**2**	**2**	**2**
6.	**1**	**2**	**2**	**2**	**3**	**3**
7.	**1**	**3**	**3**	**3**	**1**	**1**
8.	**1**	**3**	**3**	**3**	**2**	**2**
9.	**1**	**3**	**3**	**3**	**3**	**3**
10.	**2**	**1**	**2**	**3**	**1**	**2**
11.	**2**	**1**	**2**	**3**	**2**	**3**
12.	**2**	**1**	**2**	**3**	**3**	**1**
13.	**2**	**2**	**3**	**1**	**1**	**2**
14.	**2**	**2**	**3**	**1**	**2**	**3**
15.	**2**	**2**	**3**	**1**	**3**	**1**
16.	**2**	**3**	**1**	**2**	**1**	**2**
17.	**2**	**3**	**1**	**2**	**2**	**3**
18.	**2**	**3**	**1**	**2**	**3**	**1**
19.	**3**	**1**	**3**	**2**	**1**	**3**
20.	**3**	**1**	**3**	**2**	**2**	**1**
21.	**3**	**1**	**3**	**2**	**3**	**2**
22.	**3**	**2**	**1**	**3**	**1**	**3**
23.	**3**	**2**	**1**	**3**	**2**	**1**
24.	**3**	**2**	**1**	**3**	**3**	**2**
25.	**3**	**3**	**2**	**1**	**1**	**3**
26.	**3**	**3**	**2**	**1**	**2**	**1**
27.	**3**	**3**	**2**	**1**	**3**	**2**

**Table 5:** Results for Taguchi trials for average Nu, Sh, τ_w_.

Exp No.	Nu_avg_	S/N ratio	Sh_avg_	S/N ratio	τ_w_	S/N ratio
1.	1.36	2.65	1.32	2.39	2.08	−6.34
2.	1.37	2.7	1.32	2.43	1.68	−4.49
3.	1.31	2.35	1.25	1.97	1.75	−4.87
4.	1.52	3.64	1.38	2.82	3.83	−11.66
5.	1.53	3.7	1.39	2.84	2.23	−6.95
6.	1.54	3.74	1.35	2.59	3.23	−10.18
7.	1.61	4.14	1.36	2.69	6.33	−16.03
8.	1.65	4.33	1.39	2.84	2.91	−9.28
9.	1.62	4.18	1.28	2.16	5.71	−15.14
10.	0.93	−0.67	0.77	−2.22	3.43	−10.71
11.	0.97	−0.28	0.81	−1.84	1.6	−4.08
12.	0.7	−3.11	0.53	−5.44	2.79	−8.9
13.	1.25	1.91	1.21	1.66	1.02	−0.18
14.	1.28	2.12	1.24	1.86	0.76	2.33
15.	1.15	1.24	1.1	0.86	0.97	0.24
16.	1.21	1.67	1.1	0.82	2.22	−6.92
17.	1.22	1.71	1.09	0.73	1.33	−2.47
18.	1.09	0.72	0.93	−0.6	1.87	−5.45
19.	0.87	−1.21	0.81	−1.88	1.38	−2.78
20.	0.71	−2.93	0.64	−3.92	0.77	2.3
21.	0.79	−2.04	0.7	−3.1	1.11	−0.87
22.	0.43	−7.41	0.33	−9.71	2.03	−6.17
23.	0.64	−3.9	0.5	−5.97	1.06	−0.51
24.	0.46	−6.82	0.33	−9.68	1.72	−4.71
25.	1.2	1.57	1.17	1.33	0.65	3.78
26.	1.19	1.47	1.15	1.19	0.63	4.08
27.	1.2	1.62	1.15	1.25	0.65	3.79

#### Utility concept

The performance of a system is based on the quality of different characteristics. In order to get a mixed index the performance of various characteristics are then combined. Here in the present research context, final optimum settings of a BFS channel are obtained by combining all the performance functions such as average Nusselt number, Sherwood number and wall shear stress. The overall utility of the BFS channel is considered as the summation of all the utilities of performance characteristics i.e. enhancing the heat and mass transfer and reducing the wall shear stress. The overall utility function U which is a function of X_i_, represents the effectiveness measure of i_th_ performance characteristics, can be written by the following equations^39–41^:

12 $${\rm{U}}(\underset{{\rm{i}}=1}{\overset{{\rm{n}}}{{{\rm{X}}}_{{\rm{i}}}}})={\rm{U}}({{\rm{X}}}_{1},{{\rm{X}}}_{2},{{\rm{X}}}_{3}\ldots {{\rm{X}}}_{{\rm{n}}})={\rm{f}}[{{\rm{U}}}_{1}({{\rm{X}}}_{1}),{{\rm{U}}}_{2}({{\rm{X}}}_{2})\ldots {{\rm{U}}}_{{\rm{n}}}({{\rm{X}}}_{{\rm{n}}})]$$

where U_i_ (X_i_) represents the utility of i_th_ performance characteristics.

The overall utility can be written as the sum of individual utility if the performance characteristics are independent and can be written by the following expression:

13 $${\rm{U}}({{\rm{X}}}_{1},{{\rm{X}}}_{2},{{\rm{X}}}_{3}\ldots {{\rm{X}}}_{{\rm{n}}})=\mathop{\sum }\limits_{{\rm{i}}=1}^{{\rm{n}}}{{\rm{U}}}_{{\rm{i}}}({{\rm{X}}}_{{\rm{i}}})$$

The performance characteristics may be given priorities depending upon the system requirement and required weightage can be assigned to the individual utility. Then the overall utility can be rewritten as:

14 $${\rm{U}}({{\rm{X}}}_{1},{{\rm{X}}}_{2},{{\rm{X}}}_{3}\ldots {{\rm{X}}}_{{\rm{n}}})=\mathop{\sum }\limits_{{\rm{i}}=1}^{{\rm{n}}}{{\rm{W}}}_{{\rm{i}}}\,{{\rm{U}}}_{{\rm{i}}}({{\rm{X}}}_{{\rm{i}}})$$

where W_i_ is the weight allocated to the i_th_ performance characteristics and sum of all the weights of the performance characteristics must be equal to 1. For optimizing heat and mass transfer and wall shear stress for flow through a BFS channel filled with a nanofluid, the following condition has to be satisfied^39^:

15 $$\mathop{\sum }\limits_{{\rm{i}}=1}^{{\rm{n}}}{{\rm{W}}}_{{\rm{i}}}=1={{\rm{W}}}_{{\rm{Nu}}}+{{\rm{W}}}_{{\rm{Sh}}}+{{\rm{W}}}_{{{\rm{\tau }}}_{{\rm{w}}}}$$

where ${{\rm{W}}}_{{\rm{Nu}}},{{\rm{W}}}_{{\rm{Sh}}},{{\rm{W}}}_{{{\rm{\tau }}}_{{\rm{w}}}}$ are the weightage assigned to average Nusselt number, Sherwood number and wall shear stress, the response parameters of the BFS channel.

Determination of utility value for a number of performances commonly uses a preference scale and weightages are assigned to each scale to get overall utility. A logarithmic scale is used for this purpose and a preference number is set where 0 is given for the lowest acceptable quality and 9 is given for the best quality. On a logarithmic scale, the preference number (P_i_) can be calculated by the formula given below^36,40^:

16 $${{\rm{P}}}_{{\rm{i}}}={\rm{C}}\,\log \,\frac{{{\rm{X}}}_{{\rm{i}}}}{{{\rm{X}}}^{/}}$$

where X_i_ is the value of i_th_ performance characteristics (experimental trial of orthogonal array), X^**/**^ is the minimum acceptable value of i_th_ performance characteristics and C is the constant.

The value of C can be found by setting the value of preference number as 9 and X_i_ = X^*^ where X^*^ is the optimum or best value. The constant C can be expressed as:

17 $${\rm{C}}=\frac{9}{\log \,\frac{{{\rm{X}}}^{\ast }}{{{\rm{X}}}^{/}}}$$

Finally, the overall utility can be calculated by the following equation:

18 $${\rm{U}}=\mathop{\sum }\limits_{{\rm{i}}=1}^{{\rm{n}}}{{\rm{W}}}_{{\rm{i}}}\,{{\rm{P}}}_{{\rm{i}}}$$

Construction of preference scale for three different performances using Eq. (16) are shown below:

(i) Average Nusselt number:
  19 $${{{\rm{P}}}_{{\rm{i}}}|}_{{{\rm{Nu}}}_{{\rm{avg}}}}={\rm{C}}\,\log \,\frac{{{{\rm{X}}}_{{\rm{i}}}|}_{{{\rm{Nu}}}_{{\rm{avg}}}}}{0.43}$$
  where X^/^ = 0.43 = Minimum acceptable value. All the values of average Nusselt number lie in between 0.43 and 1.65, where 0.43 and 1.65 are the minimum and maximum values of average Nusselt number respectively as seen in Table 5.
(ii) Average Sherwood Number
  20 $${{{\rm{P}}}_{{\rm{i}}}|}_{{{\rm{Sh}}}_{{\rm{avg}}}}={\rm{C}}\,\log \,\frac{{{{\rm{X}}}_{{\rm{i}}}|}_{{{\rm{Sh}}}_{{\rm{avg}}}}}{0.33}$$
  where X^/^ = 0.33 = Minimum acceptable value. All the values of average Sherwood number lie in between 0.33 and 1.39, where 0.33 and 1.39 are the minimum and maximum value of average Sherwood number respectively as seen in Table 5.
(iii) Average wall shear stress

21 $${{{\rm{P}}}_{{\rm{i}}}|}_{{{\rm{\tau }}}_{{\rm{w}}{\rm{avg}}}}={\rm{C}}\,\log \,\frac{{{{\rm{X}}}_{{\rm{i}}}|}_{{\tau }_{{\rm{w}}{\rm{avg}}}}}{6.33}$$

where X^/^ = 6.33 = Minimum acceptable value. All the values of average wall shear stress lie in between 0.63 and 6.33, where 0.63 and 6.33 are the minimum and maximum value of average wall shear stress as seen in Table 5.

### Results and discussion

#### Mesh sensitivity and validation results

**Figure 3:**
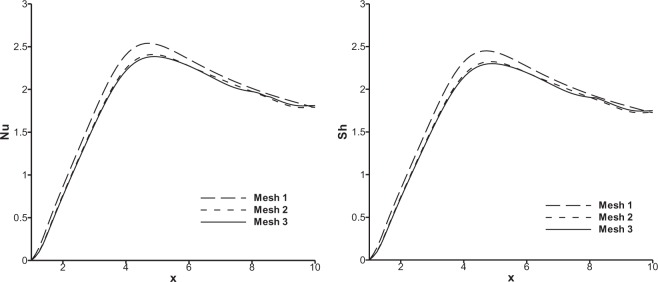
Comparison of Nusselt and Sherwood number distribution over bottom heated wall for three different meshes.

Mesh sensitivity study has been carried out in order to ensure that the simulation results are independent of discretization of the computational domain. A non-uniform mesh which is finer near the wall, is employed to capture accurately the gradients of the field variables. Three meshes, mesh 1 with 2400 elements and 2513 nodes, mesh 2 with 3300 elements and 3425 nodes and mesh 3 with 4160 elements and 4299 nodes, are considered for this purpose. Results obtained for Nusselt and Sherwood number distributions along the heated bottom wall of the channel are compared as depicted in Fig. 3. It is found that the results obtained with meshes 2 and 3 agree very closely with each other and hence mesh 2 has been chosen for further computations in the present research work. For the purpose of validation of the developed code (FORTRAN code), the results obtained in the present research are compared with the simulation results of Abu-Nada^33^ and Kanna *et al*.^34^. Reattachment length (X_r_) and local maximum Nusselt number obtained at Re = 200 for Cu nanoparticles are regarded as parameters for comparison. Table 6 shows the comparison of reattachment length obtained by the present research and those reported by Abu-Nada^33^ and Kanna *et al*.^34^ for 5% and 20% volume fractions of Cu nanoparticle. It is noted that the present numerical results compare with the results of the above authors with maximum of 8% error. Table 7 illustrates the comparison of the present results and the results of Abu-Nada^33^ for maximum local Nusselt number for 0%, 5%, 10% and 20% volume fractions of the Cu nanoparticle. It is observed that the present results agree closely with the results of Abu-Nada^40^ with 1.5% error.

**Table 6:** Comparison of reattachment length for Cu nanoparticle at Re = 200, χ = 0.05, 0.20 and Pr = 6.2.

	X_r_		Percentage error	X_r_		Percentage error
	Kanna *et al*.^41^	Present study		Abu-Nada^40^	Present study	
χ = 0.05	3.1	2.9	6.45	—	—	—
χ = 0.20	3.8	3.5	7.89	3.63	3.78	4.13

**Table 7:** Comparison of local Nu _max_ for Cu nanoparticle at Re = 200, χ = 0.00, 0.10, 0.20 and Pr = 6.2.

	Local Nu _max_		
	Abu-Nada^40^	Present study	Percentage error
χ = 0.00	2.88	2.90	0.69
χ = 0.05	3.48	3.43	1.43
χ = 0.10	3.94	3.90	1.01
χ = 0.20	5.18	5.20	0.38

#### Taguchi method

The present research work focuses on optimizing factors related to geometry, properties of working fluid and flow with an aim to achieve optimum heat and mass transfer in a BFS channel filled with alumina water/EG nanofluid. In general, researchers have used the influencing parameters as control parameters to achieve the required objectives. In the present research, six parameters such as expansion ratio (ER), Reynolds number (Re), buoyancy ratio (N), volume fraction of nanoparticles (χ), shape of nanoparticle, thermal Grashof number (Gr_T_) are taken as control parameters. Initially Taguchi optimization technique has been employed to determine the best sets of parameters to obtain optimum Nu_avg_, Sh_avg_ and τ_w avg_ separately and then utility concept is applied to get single set of parameters by satisfying all the three objective functions simultaneously by assigning suitable weightage to the objective functions. The results obtained from optimization of average Nu, Sh and τ_w_ using Taguchi method and utility concept are discussed in the following sections.

#### Analysis of S/N ratio for average Nusselt number, Sherwood number and wall shear stress

The six influential parameters considered at three levels necessitates the use of L_27_ orthogonal array giving rise to 27 trial runs in the Taguchi optimization method. These parameters at three levels are indicated by the following symbols as listed in Table 3: expansion ratio (A), Reynolds number (B), buoyancy ratio (C), nanoparticle volume fraction (D), shape of nanoparticle (E), thermal Grashof number (F). For the selected L_27_ orthogonal array, the levels of the six parameters for each trial run are shown in Table 4. Using the respective numerical values for the levels of parameters as depicted in the above table, numerical simulation results were obtained using the in-house FORTRAN computer program and from these results, average Nu, Sh and τ_w_ were computed as response for the induced variations of the six parameters. Now, using these responses, the S/N ratio were computed based on the selected performance criteria for the three objective functions. Larger the better has been chosen for the Nusselt number and Sherwood number whereas lower the better was selected for wall shear stress. Using these concepts, S/N ratios were computed for all the 27 trial cases and are shown in Table 5. Signal to noise ratio represents the preferable and unnecessary effects of the responses considered for this method. The computed responses and corresponding S/N ratios for 27 trial runs are tabulated in Table 5. Responses of S/N ratio for all the three output parameters are calculated by taking the mean of S/N ratios of the 27 trials and they are shown in response Tables 8 to 10 for average Nusselt number, Sherwood number and wall shear stress respectively. The order of effective parameters on the output parameters (average Nu, Sh and τ_w_) are shown as rank in the last row of Tables 8 to 10 where rank 1 shows the maximum influential parameter and rank 6 indicates the minimum one. The best settings of parameters for average Nu and Sh have been determined by choosing the highest value of each parameters and for average τ_w_ minimum value has to be chosen.

**Table 8:** Taguchi average S/N ratio response table for average Nusselt number.

Level	A	B	C	D	E	F
1	3.49	−0.28	−0.70	1.96	0.70	0.44
2	0.59	−0.20	1.30	1.00	0.99	0.71
3	−2.18	2.38	1.31	−1.06	0.21	0.75
Delta	5.68	2.66	2.01	3.02	0.78	0.32
Rank	1	3	4	2	5	6

**Table 9:** Taguchi average S/N ratio response table for average Sherwood number.

Level	A	B	C	D	E	F
1	2.53	−1.29	−1.96	1.66	−0.23	−0.66
2	−0.46	−1.41	0.28	0.03	0.02	−0.35
3	−3.39	1.38	0.35	−3.02	−1.11	−0.31
Delta	5.91	2.79	2.31	4.68	1.13	0.36
Rank	1	3	4	2	5	6

**Table 10:** Taguchi average S/N ratio response table for average wall shear stress.

Level	A	B	C	D	E	F
1	−9.44	−4.53	−4.66	−0.18	−6.33	−4.7
2	−4.02	−4.2	−4.54	−5	−2.12	−4.48
3	−0.12	−4.85	−4.38	−8.39	−5.12	−4.4
Delta	9.32	0.65	0.28	8.21	4.22	0.3
Rank	1	4	6	2	3	5

**Figure 4:**
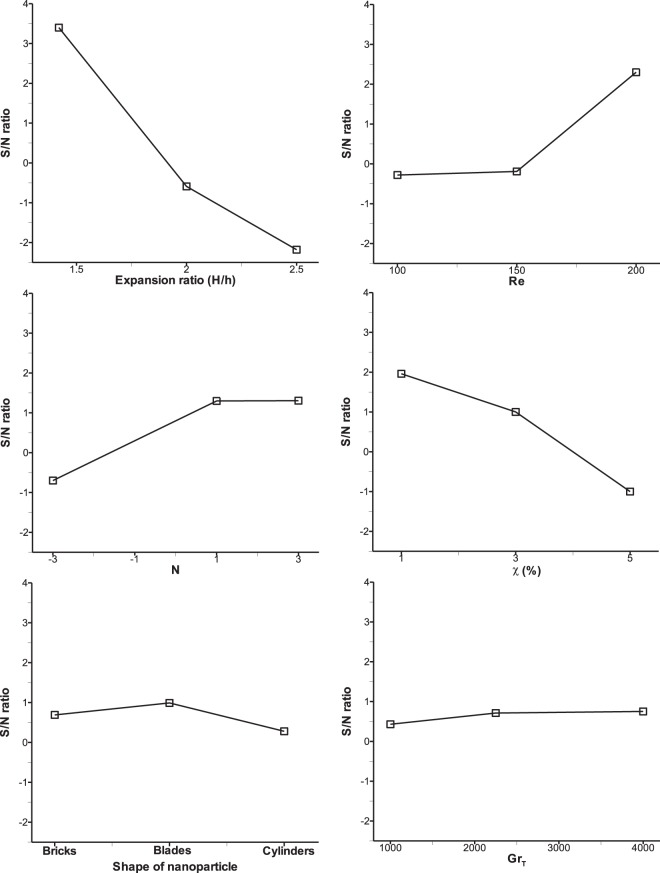
Taguchi mean S/N ratio analysis of each control factor for average Nu.

**Figure 5:**
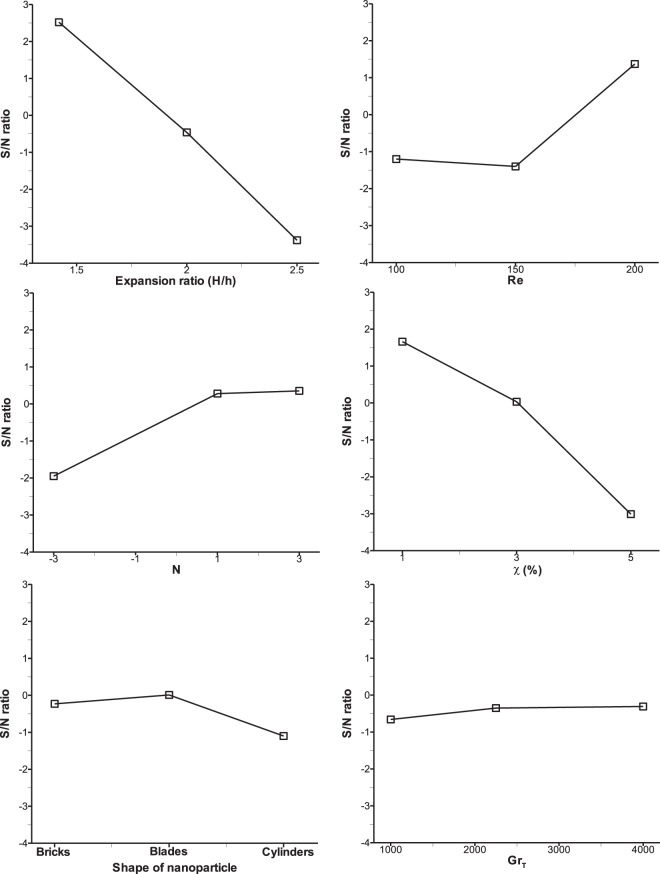
Taguchi mean S/N ratio analysis of each control factor for average Sh.

In the case of average Nu and Sh, the optimum levels of six parameters are the same and they are A1, B3, C3, D1, E2, and F3. In order to get the impact of these parameters, the S/N ratios given in Tables 8 and 9 are depicted in graphical representation as shown in Figs. 4 and 5. From these figures, one can notice the maximum slope is observed for parameter A which has the maximum influence on the responses (average Nu and Sh) and thus rank 1 is shown in Tables 8 and 9. This is on the expected line because increase in the expansion ratio, increases the depth of the step in the separation zone, which will stall the reattachment of the flow. Hence, the maximum heat and mass transfer is expected to take place at the lowest expansion ratio considered in this research. After parameter A, parameter D has the second highest slope and got 2^nd^ rank as an influential parameter. Increase in volume fraction of nanoparticle results in increase in the viscosity of the nanofluid, thus flow separation is enhanced only at the lowest volume fraction. Similarly, parameters B, C and E and F have got the subsequent ranks accordingly. Reynolds number occupies the third rank and increase in Re will always boost the inertial force that stimulates the mixing phenomena which enhances convective heat and mass transfer. The parameter F i.e. thermal Grashof number shows almost negligible slope as seen in Figs. 4 and 5 which means this parameter has the least effect on average Nu and Sh and therefore ranked 6 in Tables 8 and 9. This is to be noted that after Re, buoyancy ratio, N is found to be more influencing parameter compared to the Grashof number because thermal buoyancy force is already included in the definition of buoyancy ratio, N.

**Figure 6:**
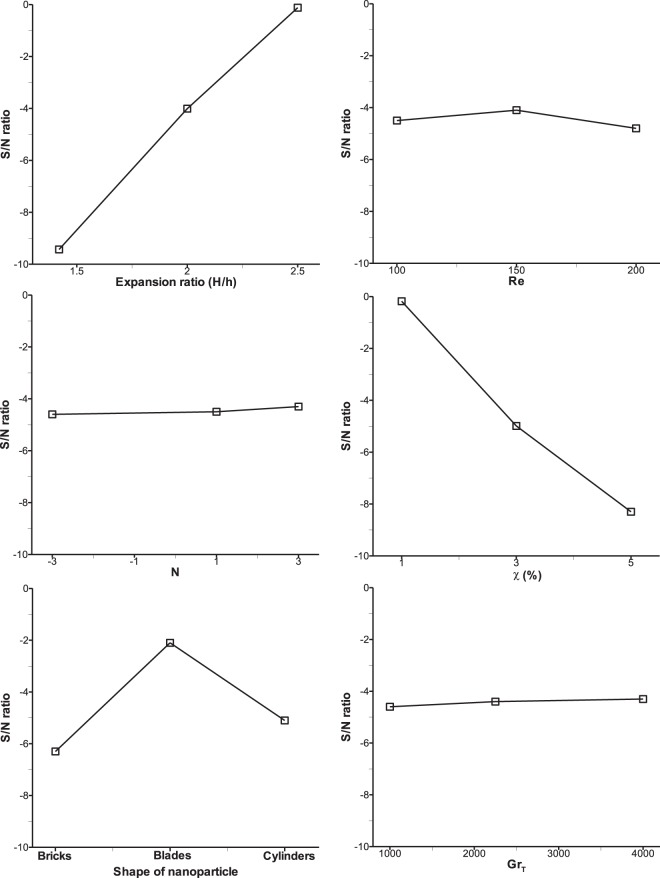
Taguchi mean S/N ratio analysis of each control factor for average τw.

For average wall shear stress, as the criterion is set as ‘lower the better’ which is different from average Nu and Sh criterion, it shows different settings of optimum parameter levels as given in response Table 10 and they are A3, B2, C3, D1, E2 and F3. The influence of various parameters is displayed in Fig. 6 where parameter A shows maximum slope of S/N ratio and thus ranked 1. This is true because with increase in expansion ratio, the velocity gradient of the fluid decreases resulting in lower wall shear stress. Similarly, parameter D occupies the 2^nd^ rank as an effective parameter which has a slope of S/N ratio next to that of parameter A. With minimum volume fraction of nanoparticle (D1), the shear stress is found to be minimum because viscosity of nanofluid attains lower value at lower volume fraction. It is very interesting to observe that parameters A and D are found to be always the most influential factors (as rank 1 and 2) for all the objective functions considered in the present research work, i.e. average Nu, Sh and τ_w_. Parameter C shows that S/N ratio slope remains almost constant irrespective of levels which means there is hardly any effect of buoyancy ratio on optimization of wall shear stress and therefore ranked 6 as seen in Table 10. Other parameters such as E, B and F fall in-between and ranked as 2, 3 and 4 respectively.

#### Analysis of variance (ANOVA)

In Taguchi optimization technique the comparative importance of control parameters on overall response are estimated as percentage contribution using analysis of variance (ANOVA). ANOVA results, consisting of degree of freedom (DF), sum of squares (SS), F ratio, P ratio and percentage contribution of each control parameter and these results for average Nu, Sh and τ_w_ are given in Tables 11 to 13. Identification of weightage of each control parameter for output response is given under F-ratio column whereas P-values give the probability used in this analysis. In order to calculate the degree of freedom and sum of squares, the following formulae are used in this analysis^23^^,^^39^:

22 $${\rm{Degree}}\,{\rm{of}}\,{\rm{freedom}}\,({\rm{DF}})={\rm{No}}.\,{\rm{of}}\,{\rm{level}}\,({\rm{L}})-1$$

23 $${\rm{Sum}}\,{\rm{of}}\,{\rm{square}}\,({\rm{SS}})=0.5(\sum {({{\rm{S}}/{\rm{N}}{\rm{ratio}}|}_{{\rm{level}}1})}^{2}+\sum {({{\rm{S}}/{\rm{N}}{\rm{ratio}}|}_{{\rm{level}}2})}^{2}+\sum {({{\rm{S}}/{\rm{N}}{\rm{ratio}}|}_{{\rm{level}}3})}^{2}-{\rm{C}}.\,{\rm{F}}.)$$

24 $${\rm{Correction}}\,{\rm{factor}}\,({\rm{C}}.\,{\rm{F}}.)=(\frac{\sum {({\rm{S}}/{\rm{N}}{\rm{ratios}})}^{2}}{{\rm{N}}})$$

where N = total number of trial experiments

25 $${\rm{Variance}}=({\rm{SS}}/{\rm{DF}})$$

26 $${\rm{F}} \mbox{-} {\rm{ratio}}=({\rm{Mean}}\,{\rm{square}}\,{\rm{of}}\,{\rm{a}}\,{\rm{parameter}}/{\rm{mean}}\,{\rm{square}}\,{\rm{error}})$$

27 $$ \% \,{\rm{contribution}}=\frac{{{\rm{SS}}}_{{\rm{i}}}}{\mathop{\sum }\limits_{{\rm{i}}=1}^{{\rm{N}}}{{\rm{SS}}}_{{\rm{i}}}}\times 100$$

**Table 11:** ANOVA for average Nusselt number.

Factors	Degree of freedom	Sum of square (ss)	Variance	F-ratios	P-values	% contribution
A	2	144.979	72.49	91.91	0.000	54.17
B	2	41.194	20.6	26.11	0.000	15.39
C	2	24.171	12.09	15.32	0.000	9.03
D	2	42.893	21.45	27.19	0.000	16.03
E	2	2.828	1.41	1.79	0.203	1.06
F	2	0.538	0.27	0.34	0.717	0.2
Error	14	11.042	0.79			
Total	26	267.646				100

**Table 12:** ANOVA for average Sherwood number.

Factors	Degree of freedom	Sum of square (ss)	Variance	F-ratios	P-values	% contribution
A	2	157.40	78.7	76.06	0.000	44.16
B	2	44.83	22.415	21.66	0.000	12.58
C	2	31.08	15.54	15.02	0.000	8.72
D	2	101.62	50.81	49.11	0.000	28.51
E	2	6.31	3.155	3.05	0.080	1.77
F	2	0.68	0.34	0.33	0.726	0.19
Error	14	14.49	1.035			
Total	26	356.40				100

**Table 13:** ANOVA for average wall shear stress.

Factors	Degree of freedom	Sum of square (ss)	Variance	F-ratios	P-values	% contribution
A	2	394.08	197.04	119.52	0.000	48.6
B	2	1.9	0.95	0.58	0.574	0.23
C	2	0.35	0.18	0.11	0.899	0.04
D	2	306.18	153.09	92.86	0.000	37.76
E	2	84.79	42.4	25.72	0.000	10.46
F	2	0.44	0.22	0.13	0.877	0.05
Error	14	23.08	1.65			
Total	26	810.82				100

The ANOVA results for average Nu is shown in Table 11. It is observed that parameter A (expansion ratio) has the major contribution (54.17%) to enhance heat transfer in BFS channel using nanofluid whereas parameter D (nanoparticle volume fraction) and B (Re) have the nominal contribution (16.03% and 15.39% respectively). Other parameters such as B, E and F have the least percentage contribution (less than 10%). From the percentage contribution it can be noticed that geometrical parameter of BFS channel plays a major role for heat transfer augmentation because the expansion ratio decides the length of flow separation which leads to heat transfer augmentation. The second most contributing factor is volume fraction of nanoparticle as inclusion of nanoparticle in the base fluid increases thermal conductivity of the working fluid which in turn leads to faster heat extraction from the heated wall. Percentage contribution of Re is almost equal to nanoparticle volume fraction and the reason behind this behavior is that increase of nanoparticle made the density of nanofluid higher than the base fluid thus increasing the inertial force. The inertial force increment helps to increase the average velocity of the fluid that convects the heat from the heated wall towards other part of the channel. Though the contribution of buoyancy effect is less than 10%, however, it forces influence on flow either by aiding or opposing thermo-solutal buoyancy forces. It can be seen from the table that shape of nanoparticle and Grashof number have negligible contributions on convective heat transfer. In the case of average Sh, the percentage contribution of parameter A was reduced compared to the one observed for average Nu as shown in Table 12. Other than the geometric parameter A, nanoparticle volume fraction occupies the major percentage contribution (28.51%) to enhance convective mass transfer. It is because the presence of nanoparticle decreases mass diffusion in nanofluid and simultaneously it increases inertial force as discussed above. As a result, there is better mixing of fluid concentration in the flow field near the bottom high concentrated wall. The effect of flow parameter B (Re) on enhancement of average Sh is slightly reduced compared to that of average Nu. Like convective heat transfer, there is hardly any contribution from thermal Grashof number and nanoparticle shape on convective mass transfer enhancement.

ANOVA results for average wall shear stress are given in Table 13. The trend of percentage contribution of each parameter differs from those observed for average Nu and Sh. It can be seen that only parameter A and D contributes the maximum (48.6% and 37.76% respectively) on mean wall shear stress. The geometric parameter (expansion ratio) of BFS channel controls the flow separation which in turn effect the velocity gradient near the bottom wall. On the same line, doping of nanoparticle effect the viscosity of the working fluid and hence it increases the wall shear stress accordingly. It is worth to notice that shape of nanoparticle also plays a nominal role as its percentage contribution is almost 10.46%. The reason behind this is that as the shape of nanoparticle changes there is a change in fluid property such as viscosity. This change in viscosity due to nanoparticle shape finally effects the wall shear stress. Because of very less percentage contributions, parameters such as B, C and F (0.23%, 0.04% and 0.05%) do not play any significant role on average wall shear stress.

**Figure 7:**
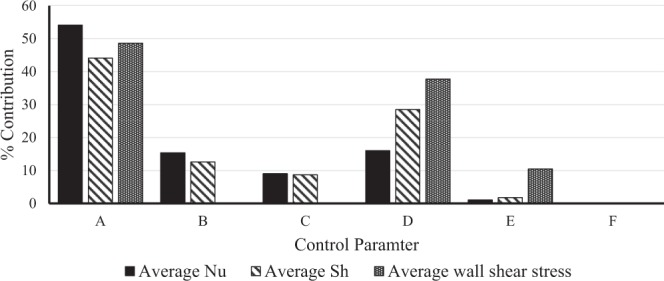
Percentage contribution of each control parameter on response parameter.

From the above discussion it has been observed that for heat and mass transfer through a BFS channel using alumina water/EG nanofluid, the mean values of Nu, Sh and wall friction are primarily controlled by the geometric parameter of the channel as shown in Fig. 7. Therefore, selection of suitable geometric parameter is crucial to achieve the required heat and mass transfer in the channel. Mixing of nanoparticle with the base fluid is the second most important aspect for convective thermo-solutal performance in BFS channel. Moreover, doping of this nanoparticle not only raises the thermal conductivity of the fluid but also increases the dynamic viscosity of the working fluid and hence it has more contribution on wall shear stress as seen in Fig. 7. The effect of shape of nanoparticle plays a vital role only for wall shear stress and thermal Grashof number has hardly shown any contribution though aiding or opposing of solutal buoyancy force represented by the parameter C has some effect on thermo-solutal performance. The Taguchi optimization results show a difference of 3.79% and 9.21% for mean convective heat and mass transfer with that of the maximum values obtained from 27 trail runs. Similarly, a difference of 33.54% is noticed for mean wall shear stress with that of the lowest value acquired from 27 numerical experiments.

#### Utility concept

Analysis of S/N ratio for average Nusselt number, Sherwood number and wall shear stress Taguchi results give the best setting of parameters to get optimum values of average Nu, Sh and τ_w_ using the respective optimization criteria independently. However, in real life engineering applications there is a need for enhancement of heat and mass transfer as well as minimization of wall friction to reduce the running cost for heat transfer fluid circulation. Therefore, in order to achieve maximum utilization of a system, optimization of multi objective functions is required and this can be achieved by using the concept of utility, which is widely used in process and quality control industry. In the present study there are three objectives i.e. maximizing the average Nu and Sh and minimizing the average τ_w._ By applying the utility concept, the utility values for all 27 trials have been computed using the expression given in Eqs. (14) to (18). As the very purpose of using nanofluid is to increase heat and mass transfer in a given thermal system, higher weightage is considered for average Nusselt number and Sherwood number whereas less weightage is given to average τ_w._ In the present analysis 40% weightage is given to average Nu, 40% weightage is given to average Sh and 20% weightage to average τ_w_. However, this weightage values can be varied depending on the demand for a given situation. The utility values and corresponding S/N ratios for 27 experimental trials are shown in Table 14.

**Table 14:** Utility values for BFS channel with nanofluid.

Exp. Trial No.	Utility value	S/N ratio
1.	7.26	17.22
2.	7.43	17.42
3.	7.18	17.12
4.	7.25	17.21
5.	7.64	17.67
6.	7.34	17.31
7.	6.96	16.85
8.	7.54	17.55
9.	6.90	16.78
10.	4.74	13.51
11.	5.48	14.77
12.	3.32	10.43
13.	7.34	17.31
14.	7.65	17.67
15.	6.97	16.86
16.	6.52	16.28
17.	6.86	16.73
18.	5.98	15.54
19.	5.29	14.46
20.	4.65	13.35
21.	4.88	13.76
22.	1.15	1.21
23.	3.61	11.15
24.	1.45	3.24
25.	7.46	17.46
26.	7.42	17.41
27.	7.46	17.45

**Figure 8:**
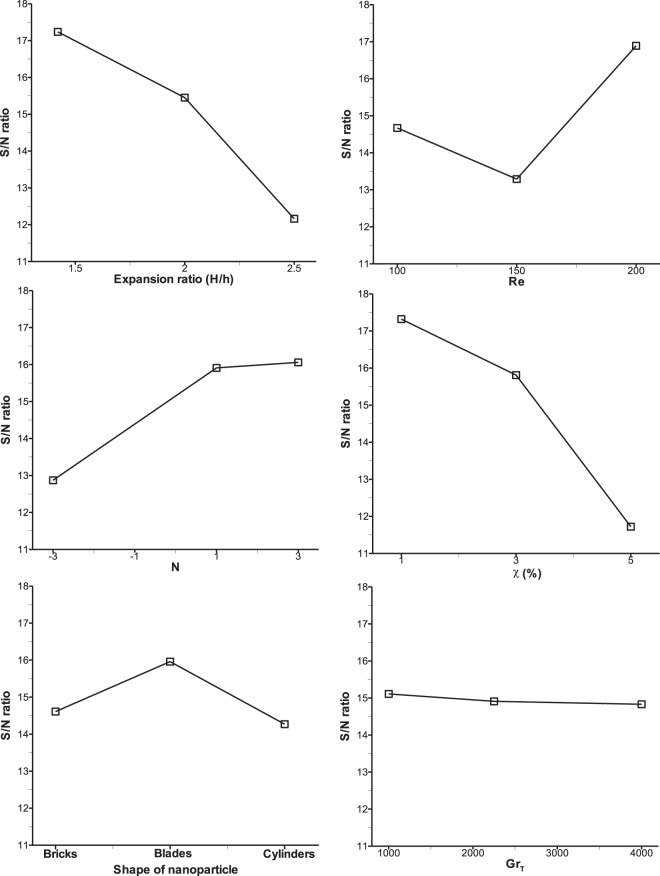
Utility S/N ratio analysis of each control factor.

The utility response results of mean S/N ratio for six control parameters at three levels are shown in Table 15. In order to achieve maximum utilization of the system by compromising mean Nu, Sh and τ_w_, an optimum setting of parameters is found as, A1, B3, C3, D1, E2 and F1. All the higher values of S/N ratio for each parameter is considered as optimum level of parameter. Figure 8 represents the slope of utility-S/N ratio for all the control parameters affecting the response. As discussed in the previous section, Grashof number is seen to be least significant as its effect is already taken into account in the buoyancy ratio, N. From the levels of parameters indicated above, it is understood that effective heat and mass transfer take place only in the narrower channel, lower volume fraction of nanoparticle in order to satisfy the wall shear stress. Aiding buoyancy force at N = 3 provides sufficient fluid convection for effective heat and mass transfer within the channel. Thus these predicted levels of parameters also support the underlying physics of the problem. After applying the utility concept, the parameters show that the optimum average Nusselt number and Sherwood number are 1.54 and 1.48 respectively whereas the optimum average wall shear stress value is 1.58 as shown in Table 16. Because some weightage is given to wall friction, the values of mean Nu and Sh decrease from those values obtained by the Taguchi method at the cost of increase of wall shear stress.

**Table 15:** Utility response table of mean S/N ratio.

Level	A	B	C	D	E	F
1	17.24	14.67	12.88	17.33	14.61	15.11
2	15.46	13.29	15.91	15.81	15.97	14.91
3	12.16	16.89	16.07	11.72	14.28	14.84
Delta	5.07	3.60	3.19	5.60	1.69	0.28
Rank	2	3	4	1	5	6

**Table 16:** Responses at weighting factors.

Sl. No	Weighting factors			Nu_avg_	Sh_avg_	τ_w_
	Nu_avg_	Sh_avg_	τ_w_			
1.	40	40	20	1.54	1.48	1.58

Analysis of variance (ANOVA) Analysis of variance (ANOVA) table has been generated using utility concept as shown in Table 17 representing the percentage contribution of each control parameter on the three objective functions, average Nu, Sh and wall shear stress related to BFS channel. From the analysis it is found that volume fraction of nanoparticle plays a prime role with 33.21% contribution. The geometric property of BFS channel i.e. expansion ratio which has the maximum percentage contribution on response parameter individually now has come down to 26.17% contribution when utility is applied. Hence, this parameter is ranked as 2 as shown in Table 15 followed by 13.06% and 12.77% contribution by parameters, B and C as rank 3 and 4 respectively. It is understood from the analysis that in order to obtain optimum performance of heat and mass transfer in a BFS channel with nanofluid, enough care must be taken on the volume percentage of nanoparticle doping in the base fluid and proper selection of expansion ratio of the channel.

**Table 17:** ANOVA for utility concept.

Factors	Degree of freedom	Sum of square (ss)	Variance	F-ratios	P-values	% contribution
A	2	119.21	59.60	15.87	0.000	26.17
B	2	59.48	29.74	7.92	0.005	13.06
C	2	58.20	29.10	7.75	0.005	12.77
D	2	151.30	75.65	20.14	0.000	33.21
E	2	14.44	7.22	1.92	0.183	3.17
F	2	0.37	0.18	0.05	0.952	0.08
Error	14	52.58	3.76	15.87	0.000	
Total	26	455.57				100

## Conclusions

The details of optimization of geometric, nanofluid properties and flow parameters for double diffusive mixed convection in a backward facing step (BFS) channel filled with nanofluid is discussed in this research article. Heat, mass and momentum transport of the flow medium is mathematically modelled using velocity-vorticity form of Navier-Stokes equations and Galerkin’s weighted residual finite element method is implemented for the numerical solution of the governing equations. As part of optimization program, three objective functions, average Nusselt number, Sherwood number and wall shear stress are considered. Taguchi method and utility concept were employed to determine the optimum levels of six parameters chosen for optimization. Using Taguchi method three different sets of parameters were obtained to achieve optimum values of average Nu, Sh and wall shear stress individually satisfying the condition of maximum Nu and Sh values and minimum wall shear stress value. In order to obtain a common set of parameters that satisfy simultaneously all the three objective functions, utility concept has been used. Analysis of variance which is the part of DoE procedure, provides information on the parameters that have maximum influence on the final performance of the objective functions. Based on the results obtained, the following conclusions were arrived at:

(i) Taguchi method predicts the same set of levels of parameters to achieve optimum values of average Nusselt and Sherwood numbers. When the parameters are used at A1B2C3D1E2F3, then the optimum value of average Nusselt and Sherwood number obtained are 1.57 and 1.52 respectively.
(ii) Expansion ratio of the channel is found to be the most influencing parameter for both Nusselt number and Sherwood number followed by volume fraction of nanoparticle and Reynolds number. However, for the case of average Nusselt number, volume fraction of nanoparticle and Re contributed at the same level, whereas for Sherwood number, nanoparticle volume fraction contributes more than that of Re. This is on the expected physics because the doped nanoparticle will have more effect on mass transfer.
(iii) An optimum wall shear stress of 0.47 is obtained for the set of parameters at A3B2C3D1E2F3. Interestingly expansion ratio also contributes the maximum to achieve this value, followed by nanoparticle volume fraction and shape of nanoparticle. Nanofluid viscosity is affected by the shape of nanoparticles and hence the wall shear stress is influenced more by the shape of nanoparticle than Reynolds number.
(iv) With the application of utility concept all the three objective functions are achieved simultaneously with 40% weightage each for Nu and Sh and 20% for wall shear stress. The optimum set of parameters are found to be A1B3C3D1E2F1 giving rise to 1.54, 1.48 and 1.58 respectively for Nu, Sh and wall shear stress.
(v) Nanoparticle volume fraction is found to contribute the maximum, followed by expansion ratio, Reynolds number, buoyancy ratio and shape of nanoparticle. Though buoyancy ratio has not appeared in Taguchi method while optimizing Nu, Sh and wall shear stress, this is found to contribute when all the three objective functions are optimized simultaneously.
